# Biscarbamate Cross-Linked Low-Molecular-Weight Polyethylenimine for Delivering Anti-chordin siRNA into Human Mesenchymal Stem Cells for Improving Bone Regeneration

**DOI:** 10.3389/fphar.2017.00572

**Published:** 2017-08-28

**Authors:** Chuandong Wang, Weien Yuan, Fei Xiao, Yaokai Gan, Xiaotian Zhao, Zhanjing Zhai, Xiaoying Zhao, Chen Zhao, Penglei Cui, Tuo Jin, Xiaodong Chen, Xiaoling Zhang

**Affiliations:** ^1^Department of Orthopedic Surgery, Xin Hua Hospital Affiliated to Shanghai Jiao Tong University School of Medicine (SJTUSM) Shanghai, China; ^2^School of Pharmacy, Shanghai Jiao Tong University Shanghai, China; ^3^Shanghai Key Laboratory of Orthopaedic Implant, Department of Orthopaedic Surgery, Shanghai Ninth People’s Hospital, Shanghai Jiao Tong University School of Medicine Shanghai, China; ^4^The Key Laboratory of Stem Cell Biology, Institute of Health Sciences, Shanghai Jiao Tong University School of Medicine and Shanghai Institutes for Biological Sciences, Chinese Academy of Sciences Shanghai, China; ^5^Department of Orthopaedics, Ruijin Hospital Affiliated to Shanghai Jiao Tong University School of Medicine Shanghai, China

**Keywords:** low-molecular-weight polyethylenimine, Chordin, human bone mesenchymal stem cells, bone morphogenetic protein-2, osteogenesis

## Abstract

Small-interfering RNA (siRNA) provides a rapid solution for drug design and provides new methods to develop customizable medicines. Polyethyleneimine 25 kDa (PEI25kDa) is an effective transfection agent used in siRNA delivery. However, the lack of degradable linkage causes undesirable toxicity, hindering its clinical application. We designed a low-molecular-weight cross-linked polyethylenimine named PEI-Et (Mn:1220, Mw:2895) by using degradable ethylene biscarbamate linkage with lower cytotoxicity and higher knockdown efficiency than PEI25kDa in delivery Chordin siRNA to human bone mesenchymal stem cells (hBMSCs). Suppression of Chordin by using anti-Chordin siRNA delivered by PEI-Et improved bone regeneration *in vitro* and *in vivo* associated with the bone morphogenetic protein-2 (BMP-2) mediated smad1/5/8 signaling pathway. Results of this study suggest that Chordin siRNA can be potentially used to improve osteogenesis associated with the BMP-2-mediated Smad1/5/8 signaling pathway and biodegradable biscarbamate cross-linked low-molecular-weight polyethylenimine (PEI-Et) is a therapeutically feasible carrier material to deliver anti-Chordin siRNA to hBMSCs.

## Introduction

When bone fracture occurs, bone marrow mesenchymal stem cells migrate to the damaged bone site, differentiate into osteoblasts, secrete extracellular matrix, and repair damaged bone (Dunlap et al., 1997). The osteogenic differentiation of human bone marrow mesenchymal stem cells is strictly regulated by various types of extracellular cytokines (Gazzerro et al., 1998). Among these cytokines, the bone morphogenetic proteins (BMPs) have received considerable attention because of their clinical applications. BMP-2 is considered one of the most potent natural stimulators of BMSC osteogenic differentiation. BMP-2 combines with types I and II receptors on the cell surface to form a complex and then activate the canonical (Smad-dependent) and non-canonical (Smad-independent) signaling pathways. BMP-2 is an important inducing factor of the osteogenic differentiation of bone marrow mesenchymal stem cells; however, the individual difference is relatively large. In addition, the clinical dosage required is 100–1000 times higher than endogenous BMPs, and complications mostly related to the off-label use of BMP-2 have been reported (Axelrad and Einhorn, 2009; Argintar et al., 2011; Kloen et al., 2012; Hinsenkamp and Collard, 2015). This finding is attributed to the biological activities of BMPs, which can be modulated extracellularly by BMP-2 inhibitors such as Chordin (Kwong et al., 2008). Chordin is a 120 kDa protein with four cysteine-rich domains of about 79 amino acids each, which binds to BMP-2, thereby preventing their interaction with BMP receptors and decreased biological activity (Piccolo et al., 1996; Larrain et al., 2000; Aspenberg et al., 2001). Chordin knockdown enhances the osteogenic differentiation of human mesenchymal stem cells and human adipose tissue-derived stromal cells, (Kwong et al., 2008; Chen et al., 2012) suggesting that Chordin in human bone mesenchymal stem cell (hBMSC) can be used as a superior therapeutic target in improving hBMSCs osteogenic differentiation and bone healing.

Small interfering RNA (siRNA) is a nucleic acid fragment containing 21–23 nucleotides that could specifically suppress the expression of a target gene via its strict design (Duan et al., 2012). Thus, siRNA provides a rapid solution for drug design and new methods to develop customizable medicines. However, the high-molecular-weight and negative charges of siRNA impede cellular uptake by target cells. Currently, the major challenge in siRNA delivery is the development of a transfection vector that exhibits biocompatibility, multifunctionality, high loading capacity, and high transfection efficiency (Xiang et al., 2012). Commercially available PEI25kDa exhibits an excellent ability to condense nucleic acid to form nanosized particles and high transfection efficiency because of the high density of cationic charges. However, the lack of degradable linkages renders the gold standard high-molecular-weight PEI (PEI25kDa) too toxic to use for clinical applications. By contrast, low-molecular-weight PEI exhibited lower cytotoxicity, but the transfection efficiency was also decreased (Fischer et al., 1999). To resolve this problem, low-molecular-weight PEI is conjugated using degradable linkers. The most common linkages used to develop biodegradable PEI derivatives included ester bonds; however, ester bonds could generate acids upon degradation, which was unfavorable for buffering the endosomal environment. By contrast, the degradation of each biscarbamate linkage generated only alcohols, CO_2_, and two amino groups (Xiang et al., 2012). The molecular weight of the cross-linking PEI-based nuclear vector is higher than 5000 Da. However, high-molecular-weight PEIs exhibit higher transfection efficiency with higher toxicity than low-molecular-weight PEIs (Fischer et al., 1999). PEI-Et which uses ethylene bis (chloroformate) as the linker to form carbamate linkage between PEI800Da molecules, exhibits higher transfection efficiency with relative low cytotoxicity in COS-7 and HeLa cell lines (Wang et al., 2012).

In previous study, we have synthesized a biodegradable small-molecular-weight PEI (MW:800Da) derivative with a carbamate linkage named PEI-Et (Mn:1220, Mw:2895). The transfection and characterization details of the polyplex formed by PEI-Et and plasmids on COS-7 and HeLa cell lines were studied as well. In the present study, PEI-Et was used to complex with siRNA to investigate the physical characteristics of the polyplex. We also examined the cytotoxicity, apoptosis, and transfection efficiency of PEI-Et on hBMSCs. The osteogenic differentiation activity of hBMSCs transfected with PEI-Et/Chordin-siRNA *in vitro* and *in vivo* was detected and identified Chordin as the most appropriate therapeutic target gene for enhancing the osteogenic differentiation of hBMScs. The knowledge of the PEI-Et/Chordin-siRNA in hBMSCs bone regenerative abilities can allow the design of novel MSC-based therapies to improve bone healing.

## Materials and Methods

### Synthesis of PEI-Et

The synthese of PEI-Et was in according to our previous study (Wang et al., 2012). First, ethylene bis (chloroformate) and branched PEI800 Da in average molecular weight (Mw) were dissolved in fresh anhydrous chloroform, respectively. The newly formed solution of ethylene bis (chloroformate) (10 mg/mL, 18.7 mL) was added dropwise to the simultaneously formed PEI solution (300 mg/mL, 3 mL) with stirring. The mixed solution was placed in an ice bath filled with nitrogen protection and allowed the reaction to continue overnight. The solvent of the sample was then evaporated under low pressure and vacuum-dried for 24 h. The sample was subsequently moved to the dialysis tube (MWCO: 1000 Da) to dissolve and dialyze against distilled water for 2 days, lyophilizing to yield a polymer. The polymer was finally stored at the temperature of -20°C for further use.

### Preparation and Characterization of PEI-Et/siRNA Polyplexes

A PEI-Et solution was added into siRNA solution in polymer-to-siRNA N/P ratios of 5, 10, 15, 25, 30, 40, 50, 75, and 100 to prepare PEI-Et/siRNA polyplexes, which were then incubated for 30 min at room temperature. A 1.0% agarose gel with 0.5 μg/mL ethidium bromide was used to load the formed siRNA nanoparticles and then subjected to electrophoresis for 30 min at 90 V. The condensing efficiency was identified by the retardation of siRNA by visualization under an UV illuminator. A Brookhaven 90Plus particle size analyzer was used to measure the particle size, the zeta potential of the nanoparticles, and the distribution of the polyplex at various N/P ratio in water. Three individual experiments were conducted to calculate the values of the zeta potentials and the particle sizes. A transmission electron microscope (TEM, JEM 2010 system JEOL, Japan) was used to observe the images of the PEI-Et siRNA polyplex.

### PEI-Et Degradation Study by Gel Electrophoresis Assay

PEI-Et degradation in phosphate buffered saline (PBS) buffer (pH = 7.4), acetate (ABS) buffer (pH 5.4), DMEM, and DMEM with 10% PBS were analyzed after a 37°C incubator. The initial concentration was 2 mg/ml. At indicated time points, the polymers were collected and analyzed for their siRNA condensation ability by agarose gel electrophoresis. The measurements were continued until the polymers non-longer showed the ability to form a polymer/siRNA complexes.

### Harvesting and Culture of hBMSCs

This study was carried out in accordance with the recommendations of Institutional Ethics Committee of Shanghai Jiao Tong University School of Medicine (SJTUSM) with written informed consent from all subjects. All subjects gave written informed consent in accordance with the Declaration of Helsinki. The protocol was approved by the Institutional Ethics Committee of Shanghai Jiao Tong University School of Medicine (SJTUSM). The hBMSCs used in the experiment were donated by patients with signed informed consent. There were four male patients aged 29, 34, 50, and 69 years. The hBMSCs separated from bone marrow aspirate in iliac bone, given their competence to adhere to the culture dish. The aspirate (2 mL) from bone marrow was inoculated in a 10 cm dish containing 20 mL of α-MEM (Hyclone, United States) supplemented with 10% fetal bovine serum (FBS; Gbico, United States) and 1% penicillin–streptomycin solution (Hyclone, United States) in a moist atmosphere containing 5% CO2 at 37°C. After 3 days of incubation, hBMSCs adhered to the culture plastic, whereas the non-adherent and hematopoietic cells were eliminated from the supernate of the dish. Once the culture reached confluence, 0.25% trypsin (Gbico, United States) was used to detach the hBMSCs, which were then stored in liquid nitrogen to reseed in 1000 cells/cm^2^ for incubation.

### Identification of hBMSC Lineage

Human bone mesenchymal stem cells (2 × 10^5^ cells) at passage 3 were cultured in monoclonal antibodies with fluorescein for 45 min at room temperature. After FACS buffer [PBS with 1% sodium azide and 10% bovine serum albumin (BSA)] was used to wash for 5 min at 1000 rpm, the stained cells were suspended again in ice-cold FACS buffer of 300 μL. The stained cells were subsequently subjected to FACS analysis (Becton Dickinson Biosciences, San Diego, CA, United States). Each sample has 1 × 104 items. FlowJo (Tree Star, United States) was used to analyze the percentage of cells showing positive signals. The antibodies used in the experiment included anti-CD29 (#555443, BD Pharmingen, United States), anti-CD34 (#555822, BD Pharmingen, United States), anti-CD44 (#555478, BD Pharmingen, United States), anti-CD45 (#340953, BD Biosciences, United States), anti-CD90 (#551401, BD Pharmingen, United States), and anti-CD105 (#323208, BioLegend, United States).

### Multi-Lineage Differential Potential

Human bone mesenchymal stem cells at passage 3 were seeded in a six-well cell culture cluster at 1 × 10^5^ cells/well incubated in BM to evaluate osteogenic differentiation. When confluence was attained, the cells were incubated in BM or OM [BM including 100 ng/mL BMP-2 (#120-02, PeproTech, United States)], with the culture medium changing every 3 days. After 21 days of osteogenic culture, alizarin red S staining (#A5533, Sigma–Aldrich, United States) was used, dissolved in 1 mL of 0.1N NaOH, and tested with a microplate reader (Infinite M200 PRO, TECAN, Switzerland) at 548 nm to quantify osteogenic differentiation. hBMSCs at passage 3 were suspended again in BM at 1 × 10^7^ cells/mL for chondrogenic differentiation assays. Three drops containing a 10 μL cell suspension were carefully added into a 12-well plate. After adhesion for 2 h, BM or a chondrogenic medium was used [BM including 10 ng/mL TGF-beta1 (recombination human transforming growth factor-beta 1; #100-21, PeproTech, United States), 0.5 mg/mL BSA (Bovine Serum Albumin #A1933, Sigma, United States), 1× ITS (#41400-045, Invitrogen, United States), 4.7 μg/mL linoleic acid (#L1376, Sigma, United States), 37.5 μg/mL ascorbic acid (#A4403, Sigma, United States), and 100 nM dexamethasone (#D4902, Sigma, United States)], with culture medium changed every 3 days. After chondrogenic culture for 28 days, the micromass was ready for Alcian Blue (#A5268, Sigma, United States) staining and paraffin sectioning. hBMSCs at passage 3 were seeded in a six-well plate at 1 × 105 cells/well, cultured in BM for adipogenic differentiation assays. When confluence was attained, the cells were cultured in adipogenic medium or BM [BM including 500 nM dexamethasone, 50 mM indomethacin (#I7378, Sigma, United States), 0.5 mM isobutylmethylxanthine (#I7018, Sigma, United States), and 10 mg/mL insulin (#I3536, Sigma, United States)], with the medium changed every 3 days. After adipogenic culture for 14 days, the cells were ready for Oil red-O (#O0625, Sigma–Aldrich) staining.

### MTT Assay

MTT (#M5655, Sigma, United States) assay was used to determine the cytotoxicity of the formed polyplexes of PEI-Et and siRNA to hBMSCs. hBMSCs at passage 3 were seeded and cultured in 96-well plates at 1 × 104 cells/well for 24 h. The culture medium was substituted by media containing various concentrations of PEI-Et or polyplexes ranging from 0.5 to 100 μg/mL, with different N/P ratios ranging from 5 to 100. After incubation at 37°C under an air atmosphere containing 5% CO2 for 4 h, the culture medium was then substituted by 200 μL/well MTT reagent (0.5 mg/mL MTT dissolved in a complete culture medium), added to every well, and incubated under the same condition for another 4 h. The MTT reagent without reaction was removed, and violet crystals were added and dissolved in every well containing 200 μL of dimethyl sulfoxide. A microplate reader (Infinite M200 PRO, TECAN, Switzerland) was used to measure absorbance at a wavelength of 570 nm with a reference wavelength of 630 nm. The formula for cell viability calculation was as follows: [(OD570 - OD630) test/(OD570 - OD630) control] × 100%.

### Real-time PCR

TriPure Isolation Reagent (#93956520, Roche, Switzerland) was used for the isolation of the total RNA of hBMSCs in accordance with the manufacturer’s instructions. A PrimeScript RT Master Mix cDNA Synthesis Kit (#RR036A-1, Takara, Japan) was used for the reverse transcription of RNA samples (1 μg) to obtain the first-strand cDNA. LightCycler480 (Roche, Switzerland) system with SYBR Premix ExTaqTM reagent (#RR420a, Takara, Japan) was used in accordance with the manufacturer’s instructions to measure real-time PCR. The real-time PCR conditions were set as follows: denaturation at 95°C for 30 s, 50 cycles at 95°C for 10 s, and 60°C for 30 s. β-Actin was used as the housekeeping gene. The sequences of the gene primers used were as follows: Chordin, forward primer: TTCGGCGGGAAGGTCTATG, reverse primer: ACTCTGGTTTGATGTTCTTGCAG; Sp7 transcription factor (OSX), forward primer: CCTCTGCGGGACTCAACAAC, reverse primer: AGCCCATTAGTGCTTGTAAAGG; osteocalcin (OCN), forward primer: GAAGCCCAGCGGTGCA, reverse primer: CACTACCTCGCTGCCCTCC; collagen, type I, alpha1(Col1a1), forward primer: GAGGGCCAAGACGAAGACATC, reverse primer: CAGATCACGTCATCGCACAAC; β-actin, forward primer: CATGTACGTTGCTATCCAGGC, reverse primer: CTCCTTAATGTCACGCACGAT.

### Chordin Knockdown and Cellular Uptake Efficiency

On the basis of the sequence of the human Chordin (Gene Accession Number: NM_003741), Chordin-specific siRNA was used to knock down Chordin expression. The designed Chordin-specific siRNA targets the sequence 5′-CAGGTGCACATAGCCAACCAA-3′. The sense and antisense sequences of the siRNA were r(GGUGCACAUAGCCAACCAA)dTdT and r(UUGGUUGGCUAUGUGCACC)dTdG, respectively. The sequence of siRNA was synthesized by Shanghai GenePharma Co., Ltd. The transfection efficiency of PEI-Et was assessed by the FITC-negative control siRNA (GenePharma, Shanghai) with fluorescein. The FITC-negative control siRNA with fluorescein is not homologous to any known gene, ensuring against non-specific cellular gene expression caused by the introduction of the oligonucleotide into the cells. hBMSCs were incubated under the same experimental conditions and transfected with FITC-negative control with fluorescein at various N/P ratios. After incubation for 4 h, FACS analysis (Becton Dickinson Biosciences, San Diego, CA, United States) with FlowJo flow cytometry software (Tree Star, United States) was conducted to measure the proportion of transfected cells. The hBMSCs were then transfected using a negative control with cy3-fluorescein at various N/P ratios. After incubation for 1 h, Hoechst33342 (Invitrogen) and Lyso-Tracker^TM^ Green DND-26 were used to label the nucleus and the lysosome of the hBMSCs. Laser confocal microscopy (Cell Observer, ZEISS, Germany) was also conducted.

### Apoptosis and JC-1 Staining

Human bone mesenchymal stem cells at passage 3 were incubated with a density of 1 × 105 cells/well onto a six-well plate, cultured in complete BM. After 24 h culture, the complete medium was substituted by 1 mL of Opti-MEM (#31985062, Life Technologies, United States) containing polyplexes with various N/P ratios. The cells were harvested and then washed with cold PBS after incubation for 4 h. An apoptosis analysis kit (#V13241, Life Technologies, United States) was used in accordance with the manufacturer’s instructions to stain the hBMSCs. The FlowJo software (Tree Star, United States) was used to quickly analyze the flow cytometry of the stained cells. A JC-1 analysis kit (#C2005, Beyotime, China) and a LIVE/DEAD Viability/Cytotoxicity kit (#L-3224, Life technologies, United States) were used in accordance with the manufacturer’s instructions to stain the cells after transfection with different polyplexes. Laser confocal microscopy (Cell Observer, ZEISS, Germany) was immediately used to observe the stained hBMSCs.

### Western Blot Analysis

A lysis buffer containing 50 mM Tris–HCl at pH 7.4, 150 mM NaCl, 0.1% SDS, 1% Nonidet P-40, 1 mM PMSF, and protease inhibitor cocktail (10 mg/mL leupeptin, 10 mg/mL pepstatin A, and 10 mg/mL aprotinin) was used to lyse the hBMSCs on ice for 30 min for Western blot analysis. The protein parts were collected by centrifugation under the condition of 15,000 *g* at 4°C for 10 min, subjected to 10% SDS–PAGE, and finally transferred onto nitrocellulose membranes. The transferred membranes were blocked by 5% BSA and cultured with specific antibodies at 4°C overnight. An enhanced chemiluminescence detection system (#WBKLS0500, Millipore, Billerica, MA, United States) was used in accordance with the manufacturer’s instructions for visualization after adding the horseradish peroxidase–labeled secondary antibody. The primary antibodies used were as follows: Runx2 mouse mAb (1:1000, #ab76956, Abcam, United States), Smad1 Rabbit mAb (1:1000, #6944, Cell Signaling Technology, United States), phospho-Smad1/5/8 Rabbit pAb (1:1000, #4086, Cell Signaling Technology, United States), and GAPDH Rabbit mAb (1:1000, # 2118, Cell Signaling Technology, United States).

### ALP Analysis and Staining

For ALP analysis, hBMSCs were collected using 0.25% trypsin and then repeatedly frozen and thawed thrice. The cell lysate supernatant was extracted by centrifugation at 12000 rpm for 10 min at 4°C. PNPP was determined by adding 100 μL of DEA (50 mmol/L glycine and 1 mmol/L MgCl2 at pH 10.5), 50 μL of PNPP solution (#N7653, Sigma, United States), and 50 μL of cell lysate supernatant into the 96-well plates and then incubated for 15 min at 37°C. After incubation, 400 μL of 1N NaOH was added into each well to stop the reaction, and the absorbance was measured at 405 nm. The quantity of p-nitrophenol liberated from the substrate was determined by comparison to a standard curve. The total protein concentration was measured by BCA. Subsequently, 200 μL of solution A (#23228, Pierce, United States), 4 μL of solution B (#1859078, Pierce, United States), and 20 μL of cell lysate supernatant were added into each well and then incubated for 30 min at 37°C; the absorbance was measured at 562 nm. ALP activity was normalized to the number of total protein and expressed as nmol nitrophenol product/minute/absorbance. Alkaline phosphatase staining was performed using alkaline phosphatase staining reagent (Shanghai Hongqiao Le Xiang Institute of Biomedical, China) in accordance with the manufacturer’s instructions.

### Femoral Monocortical Defect Model

Animal studies were conducted according to procedures and principles approved by the Animal Care Committee of Shanghai Jiao Tong University, Shanghai, China. The femur monocortical defect model used is a simplified stable fracture model, described previously (Yamamoto et al., 2015; Hu and Olsen, 2016). Eight-week-old BALB/c-nu male mice were used for all experiments. Mice were placed under general anesthesia by i.p. injection of 100 mg/kg ketamine and 10 mg/kg xylazine. The lateral aspect of right tibia was exposed and carefully cleared of overlying soft tissues while preserving the PO. A monocortical osseous hole (0.8 mm diameter) was created on the anterior surface of the femoral crest using a round burr attached to a dental drill. Irrigation with saline was used to remove bone dust and fragments. hBMSCs (5 × 10^4^ cells per sample) isolated from bone non-union patients at passage 3 resuspended in a mixture of medium and Matrigel (#356234, BD Bioscience, United States) and then transplanted to the osseous hole. The soft-tissue wound was closed by separately suturing the muscle and skin layers with 5-0 absorbable gut suture. After 1 month, tibiae were isolated, fixed overnight in 10% neutral buffered formalin, and kept in 70% ethanol until analyzed using a μCT35 system (SCANCO Medical) with a spatial resolution of 5 μm. Sagittal image sections of injured tibiae were used to perform 3D histomorphometric analysis. We defined the regions of interest as (i) the hole region between the interrupted cortical bone ends, (ii) injured BM, and (iii) periosteal callus outside the hole (**Supplementary Figure S2**). Old bone fragments remaining from the drilling were excluded from the regions of interest. A total of 96–100 consecutive images (about 0.672–0.7 mm in length) were used for 3D reconstruction and analysis, covering most of the injured region and periosteal callus. Structural parameters (3D) included BMD, total tissue volume (TV), trabecular BV per TV (BV/TV), Tb.N, Tb.Th, and Tb.Sp. Fixed samples were decalcified in 0.5 M EDTA (pH 8.0) for 14 days and embedded in paraffin for H&E staining.

### Immunofluorescence Assay

Immunostaining was performed using a standard protocol. Sections were incubated with primary antibody against OCN Rabbit pAb (1:200, #ab93876, Abcam, United States) overnight at 4°C, primary antibodies were detected using FITC-conjugated anti-Rabbit IgG secondary antibody. After the final wash, the nuclei was counterstained by DAPI (1:1000 #D1306, Life technologies, United States) in PBS for 10 min before imaging. The stained sections were immediately observed through laser confocal microscopy (Cell Observer, ZEISS, Germany).

### Statistical Analysis

All numerical data were expressed as means ± SD for the number of experiments. SPSS version 13.0 was used for all statistical analyses. Student’s *t*-test were used for two sample comparisons and ANOVA with Tukey’s *post hoc* test for multiple comparisons in statistical analysis, and ^∗^*P* < 0.05 and ^∗∗^*P* < 0.01 were considered statistically significant.

## Results

### Characterization of PEI-Et/siRNA Polyplexes

**FIGURE 1:**
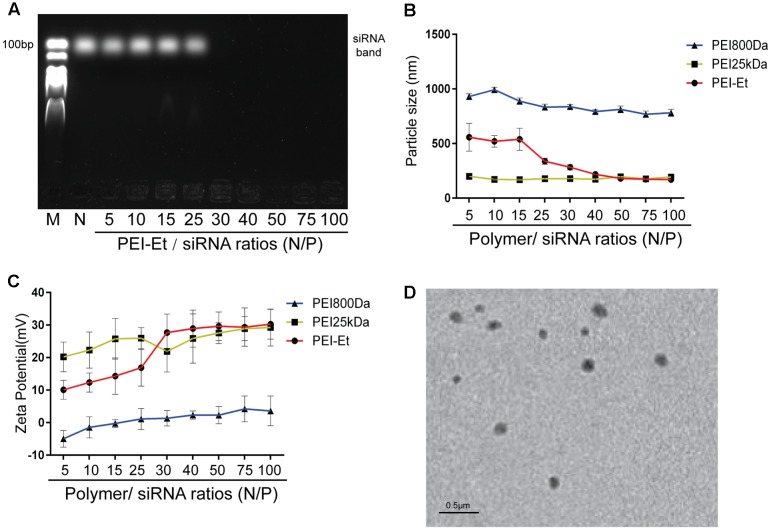
Characterization of PEI-Et/siRNA polyplex. **(A)** Agarose gel electrophoresis results of the PEI-Et/siRNA polyplex. Polyplex formed from polymer to siRNA at N/P ratios of 5, 10, 15, 25, 30, 40, 50, 75, and 100. **(B)** Particle size of polymer/siRNA polyplex at different N/P ratios. Data are presented as mean ± SD of three independent experiments, *N* = 3. **(C)** Particle size of polymer/siRNA polyplex at different N/P ratios. Data are presented as mean ± SD of three independent experiments, *N* = 3. **(D)** Morphology of the complexes are measured with transmission electron microscope (TEM).

Agarose gel electrophoresis determines the siRNA condensing efficiency of PEI-Et. As shown in **Figure 1A**, when the N/P ratio of PEI-Et to siRNA exceeded 30, siRNA was delayed at the well. Thus, this polymer could condense siRNA completely. Dynamic light scattering was used to measure the sizes of the formed nanoparticles. As shown in **Figure 1B**, the particle sizes of different polymer (PEI800Da, PEI25kDa and PEI-Et)/siRNA complexes with various N/P ratios were also examined. The particle size of PEI-Et polyplexes was around 180 nm with a variety of N/P ratios from 40 to 100 which was comparable with that of PEI25kDa. This result was consistent with the morphology of PEI-Et/siRNA polyplexes under TEM (**Figure 1D**). However, when the polymer/siRNA N/P ratios were increased from 5 to 100, the PEI800Da/siRNA complexes formed particles with sizes above 800 nm. PEI800Da exhibited variable zeta potentials with charges ranging from -4.95 ± 2.56 to 3.62 ± 4.56 mV. PEI-Et and PEI25kDa retained constant positive charges around 30 mv when N/P ratios above 40 (**Figure 1C**).

### Cytotoxicity of PEI-Et and PEI-Et/siRNA Polyplexes to hBMSCs

**FIGURE 2:**
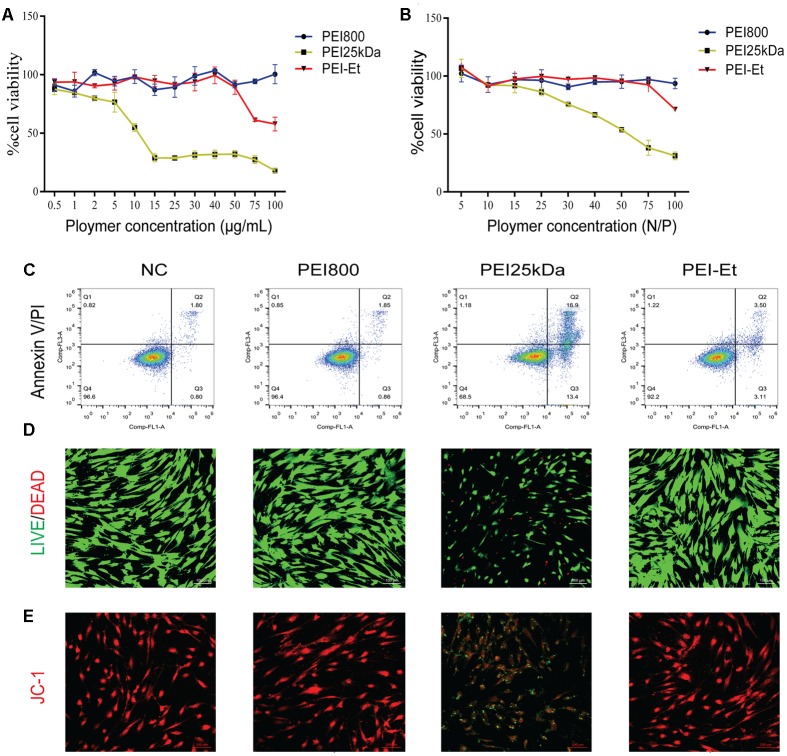
Cytotoxicity of PEI-Et and PEI-Et/siRNA polyplexes to hBMSCs. **(A)** Cytotoxicity of different transfection vectors at concentrations ranging from 0.5 to 100 μg/mL. Data represents the means ± SD of three independent experiments, *N* = 3. **(B)** Cytotoxicity of different transfection polyplexes at different N/P ratios. Data represent the means ± SD of three independent experiments, *N* = 3. **(C)** Apoptosis of hBMSCs measured with Annexin V/PI staining after transfection with different polyplexes. Q1: dead cells/cellular debris, Q2: late apoptotic/necrotic cells, Q3: early apoptotic cells, Q4: normal cells. **(D)** Cell viability of hBMSCs measured using the LIVE/DEAD Viability/Cytotoxicity Assay Kit after transfection with different polyplexes. Green: normal cells; Red: dead cells. The scale is 100 μm. **(E)** Mitochondrial membrane potentials of hBMSCs measured using JC-1 stain. Red: normal mitochondrial membrane potential; Green: reduced mitochondrial membrane potential. The scale is 100 μm.

As for the therapeutic application of a synthetic nucleic acid carrier, cytotoxicity has drawn considerable attention. The cytotoxicity of PEI-Et on hBMSCs was first evaluated by MTT assay. The identification and characterization of hBMSCs were evaluated (**Supplementary Figure S1**). The cytotoxicity levels of the various concentrations of PEI-Et or PEI25kDa, as well as different ratios of PEI-Et/siRNA or PEI 25 kDa/siRNA polyplexes, were examined after incubation with hBMSCs for 4 h. When the concentration of PEI-Et polymer exceeded 50 μg/ml, the cell viability of hBMSCs decreased. By contrast, when the concentration of polymer reached 15 μg/L, the cell viability of PEI25kDa-treated hBMSCs markedly decreased to 20% of its original value (**Figure 2A**). Meanwhile, PEI-Et/siRNA polyplexes with N/P ratios ranging from 5:1 to 75:1 exerted no negative effects on the cell viability of hBMSC (**Figure 2B**). Nevertheless, when N/P exceeded 20, the cytotoxicity of the PEI25kDa/siRNA polyplexes markedly increased (**Figure 2B**).

Apoptosis was examined after incubation for 4 h with hBMSCs, indicating that the PEI25kDa/siRNA group exhibited higher apoptotic efficiency, compared with the PEI-Et/siRNA groups (**Figure 2C**). The PEI-Et/siRNA groups hardly induced hBMSC apoptosis; meanwhile, the viable cells were 92.2%. No significant difference between the PEI-Et/siRNA and the PEI800/siRNA groups was indicated, as shown in **Figure 2C**. After the culturing for 4 h, most of the apoptotic cells induced by PEI25kDa/siRNA were at the early stage of apoptosis. After polyplexes with hBMSCs underwent incubation for 4 h, cell viability detection (LIVE/DEAD Viability/Cytotoxicity Assay Kit) showed that cell viability markedly decreased in the PEI25kDa/siRNA group compared with the PEI-Et/siRNA and PEI800 groups (**Figure 2D**). The low apoptotic cell ratio in the PEI-Et/siRNA group was subsequently investigated. The polycation polymer compound with a highly positive charge could have influenced the potential of the normal intracellular membrane. This phenomenon could also be the main reason for the occurrence of cell apoptosis and reduction in cell viability. In addition, the potentials of the mitochondrial membrane within the 4 h incubation of the hBMSCs with PEI25kDa/siRNA and PEI-Et/siRNA polyplexes were detected by JC-1. Results indicated that mitochondrial membrane potentials in the PEI-Et/siRNA group were normal, whereas mitochondrial membrane potentials in the hBMSCs with PEI25kDa/siRNA polyplexes group was considerably reduced (**Figure 2E**).

### Cellular Uptake Efficiency of PEI-Et in Delivering siRNA into hBMSCs

**FIGURE 3:**
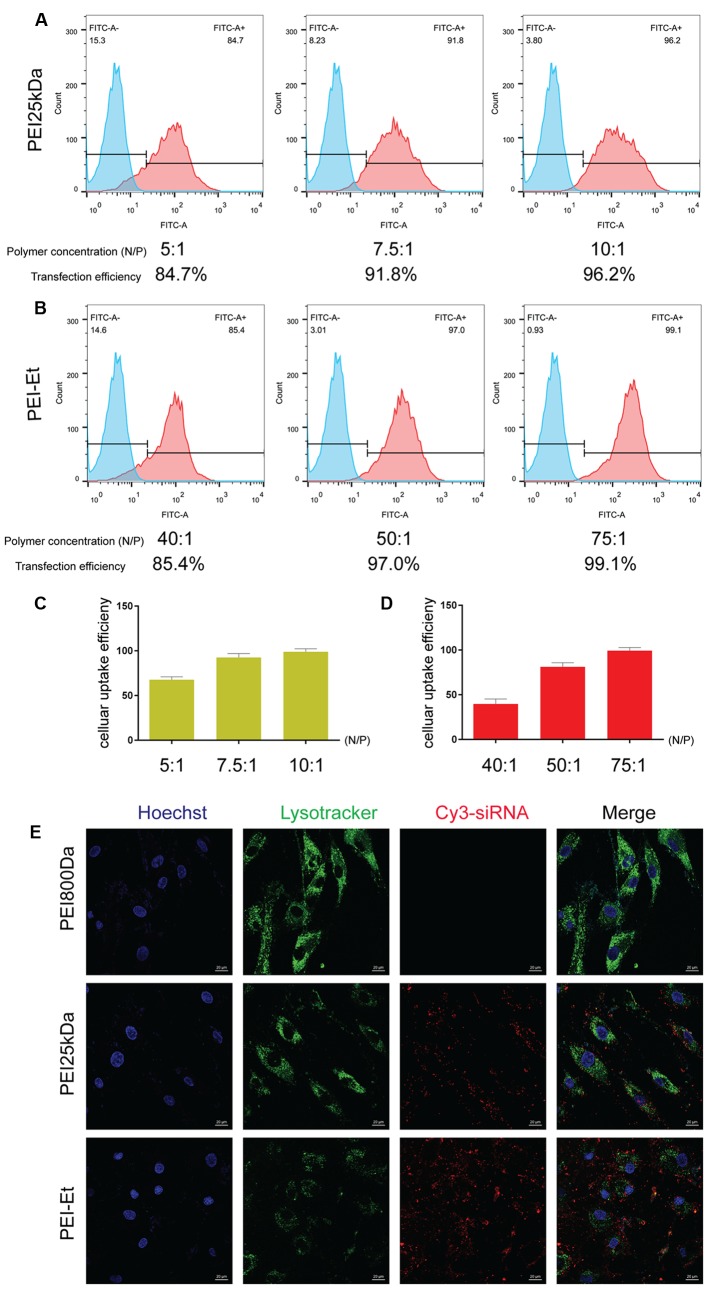
Cellular uptake efficiency of PEI-Et in delivering siRNA into hBMSCs. **(A)** Representative flow cytometry images of cellular uptake efficiency of PEI25kDa/siRNA polyplexes at different N/P ratios (5:1, 7.5:1, and 10:1). **(B)** Representative flow cytometry images of cellular uptake efficiency of PEI-Et/siRNA polyplexes at different N/P ratios (40:1, 50:1, and75:1). **(C)** Quantitative analysis cellular uptake efficiency of PEI25kDa/siRNA polyplexes at different N/P ratios (5:1, 7.5:1, and 10:1). Data are presented as mean ± SD of three independent experiments, *N* = 3. **(D)** Quantitative analysis cellular uptake efficiency of PEI-Et/siRNA polyplexes at different N/P ratios (40:1, 50:1, and 75:1). Data are presented as mean ± SD of three independent experiments, *N* = 3. **(E)** Cellular uptake of PEI800/Cy3-siRNA, PEI25kDa/Cy3-siRNA and PEI-Et/Cy3-siRNA polyplexes measured by laser confocal microscopy. Red: polyplexes; Blue: cell nucleus, Green: lysosome. The scale is 20 μm.

The efficiencies of PEI-Et and PEI25kDa in delivering FITC-siRNA into hBMSCs were compared. The hBMSCs were transfected with PEI-Et/FITC-siRNA polyplexes and PEI25kDa/FITC-siRNA polyplexes at different N/P ratios, and flow cytometry was used to measure their efficiencies. At the optimal N/P ratios of 75:1 and 10:1, the transfection efficiency of the cells treated with PEI-Et- and PEI25kDa-delivered FITC-siRNAs was 99.1 and 96.2%, respectively. These results showed that with increased N/P ratio, the PEI-Et/siRNA and PEI25kDa/siRNA polyplexes exhibited increased transfection efficiency as well (**Figures 3A–D**).

The cellular uptake of the PEI-Et/siRNA and PEI25kDa/siRNA polyplexes was examined. After 1 h of cultivation with hBMSCs, confocal microscopy was conducted to evaluate the PEI-Et/siRNA (N/P 100:1) and PEI25kDa/siRNA (N/P 20:1) polyplexes. Lysosome was labeled using Lyso-tracker^TM^ DND-26 (green), whereas siRNA was labeled using Cy3 (red). The results indicated that the PEI-Et/siRNA and the PEI 25 kDa/siRNA polyplex groups exhibited high cellular uptake by hBMSCs (**Figure 3E**).

### Chordin Knockdown Efficiency by PEI-Et/Chordin-siRNA Polyplexes

**FIGURE 4:**
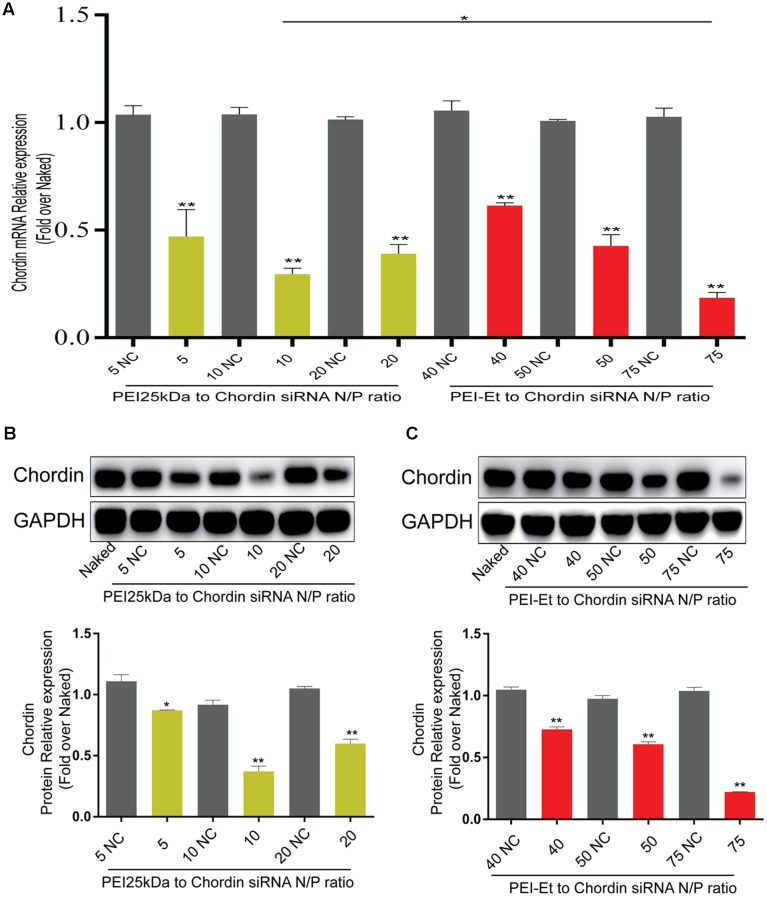
Chordin knockdown by PEI-Et/Chordin-siRNA polyplexes. **(A)** Chordin mRNA expression measured by real-time PCR after transfection with PEI25kDa/Chordin-siRNA polyplexes and PEI-Et/Chordin-siRNA polyplexes at different N/P ratios. **(B)** Chordin protein expression measured by Western blot analysis after transfection with PEI25kDa/Chordin-siRNA polyplexes different N/P ratios. Representative images of three repeated experiments were shown. **(C)** Chordin protein expression measured by Western blot analysis after transfection with PEI-Et/Chordin-siRNA polyplexes different N/P ratios. The Chordin protein level was normalized to GAPDH. Data are represents as means ± SD of three independent experiments. ^∗^*P* < 0.05, ^∗∗^*P* < 0.01.

To evaluate the Chordin knockdown efficiency of PEI-Et in delivering Chordin-siRNA, hBMSC transfection was conducted by the antisense and nonsense Chordin-specific siRNAs that were packed into polyplexes with PEI-Et and PEI25kDa at different N/P ratios. After cultivation for 3 days, Chordin mRNA expression was evaluated by real-time PCR and Western blot analysis. The cells treated with PEI-Et-delivered antisense Chordin-siRNA, namely, the PEI-Et/Chordin-siRNA group, exhibited Chordin silencing rates of 38.57, 57.30, and 81.44% at N/P ratios of 40:1, 50:1, and 75:1, respectively. Meanwhile, the cells treated with PEI25kDa-delivered Chordin-siRNA, namely, the PEI25kDa/Chordin-siRNA group, showed Chordin silencing rates of 52.91, 70.48, and 60.99% at N/P ratios of 5:1, 10:1, and 20:1, respectively (**Figure 4A**). In the PEI25kDa/Chordin-siRNA group, the reduction of Chordin silencing rate from the N/P ratio of 20:1 to 10:1 may be attributed to the cytotoxicity of polyplexes to hBMSCs. Similar to the expression level of Chordin mRNA, the expression levels of Chordin protein in the PEI-Et/Chordin-siRNA groups were reduced by 27.26, 39.35, and 77.85% at the N/P ratios of 40:1, 50:1, and 75:1, respectively (**Figure 4B**). Then, the expression of Chordin protein in PEI25kDa/Chordin-siRNA groups was reduced by 12.78, 63.81, and 40.09% at the N/P ratios of 5:1, 10:1, and 20:1, respectively (**Figure 4C**).

### Osteogenic Gene Expression in hBMSCs Transfected with PEI-Et/Chordin-siRNA Polyplexes

**FIGURE 5:**
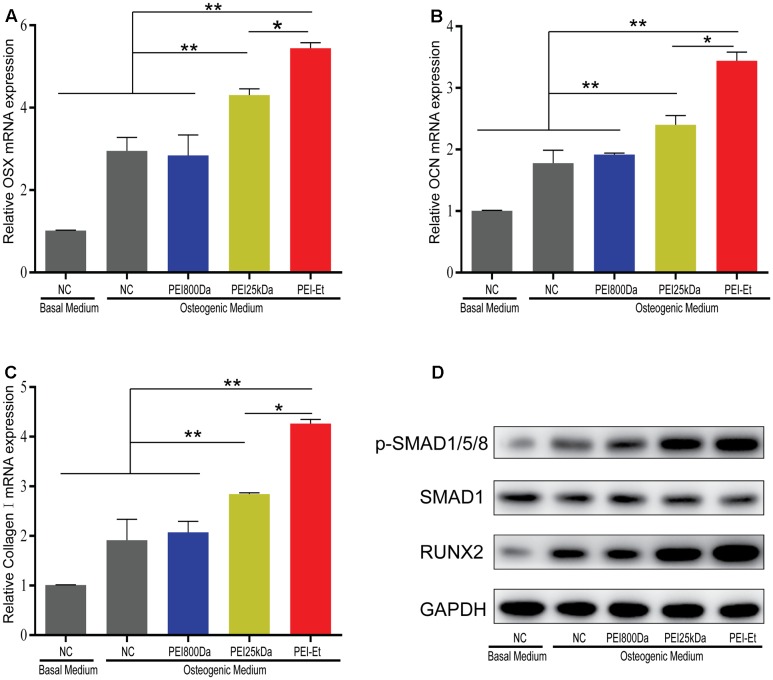
Osteogenesis gene expression of hBMSCs transfected with PEI-Et/Chordin-siRNA polyplexes. The mRNA expression levels of OSX **(A)**, OCN **(B)**, and Col1a1 **(C)** were measured by real-time PCR at 7 days after transfection with different polyplexes. **(D)** The expression levels of RUNX2 and p-SMAD1/5/8 complexes were measured by Western blot analysis. RUNX2 and p-SMAD1/5/8 protein levels were normalized to GAPDH and SMAD1, respectively. Representative images of three independent experiments were shown. Data are presented as the means ± SD of three independent experiments. ^∗^*P* < 0.05, ^∗∗^*P* < 0.01.

Transfection with Chordin-specific siRNA was conducted after 7 days of osteogenic differentiation. The osteogenesis-related genes including osterix (OSX), osteocalcin (OCN) and type I collagen (Col1a1) were then measured by real-time PCR to test the differentiation potential of hBMSCs. The mRNA expression levels of OSX, OCN, and Col1a1 increased in the PEI25kDa/Chordin-siRNA and PEI-Et/Chordin-siRNA groups compared with that in the negative control (naked siRNA) and the PEI800/Chordin-siRNA groups. The expressions levels of OSX, OCN, and Col1a1 were markedly higher in the PEI-Et/Chordin-siRNA groups than in the PEI25kDa/Chordin-siRNA groups (**Figures 5A–C**). In addition, the expression levels of these genes in the hBMSCs transfected with the PEI-Et/Chordin-siRNA groups were 1.12, 1.19, and 1.43 times higher than those in the PEI25kDa/Chordin-siRNA groups, respectively. We supposed that the suppression of Chordin expression could enhance not only the biological activity of BMP-2 but that of its signaling pathway as well. Significant signal molecules in the signaling pathway of BMP-2 included Smad1/5/8 and Runx2, which could have been involved in hBMSC osteogenic differentiation. When the signaling pathway of BMP-2 was enhanced, the expression of phospho-smad1/5/8 and Runx2 increased accordingly. Western blot analysis indicated that compared with those in the negative control (naked siRNA) groups with osteogenic medium and PEI800/Chordin-siRNA, the expression levels of phospho-smad1/5/8 and Runx2 were enhanced in the PEI25kDa/Chordin-siRNA and the PEI-Et/Chordin-siRNA groups. These results indicated that when the expression of the BMP-2 inhibitor Chordin was reduced, the signaling pathway of BMP-2 enhanced (**Figure 5D**). Furthermore, the expression levels of phospho-smad1/5/8 and Runx2 were higher in the PEI-Et/Chordin-siRNA groups than that in the PEI25kDa/Chordin-siRNA groups, indicating that PEI-Et/Chordin-siRNA exhibited a greater potential than PEI25kDa/Chordin-siRNA in promoting hBMSC osteogenic differentiation.

### Chordin Knockdown Improves the Osteogenic Capacity of hBMSCs

**FIGURE 6:**
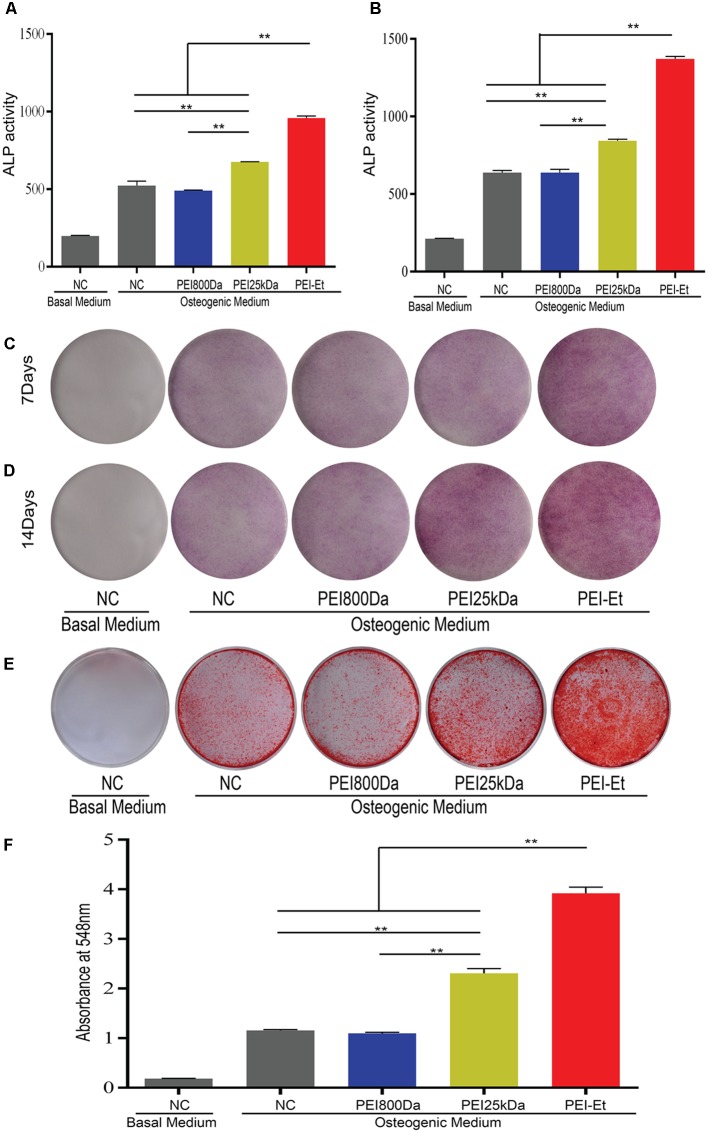
Chordin knockdown improving the osteogenic capacity of hBMSCs. ALP activity measured at 7 **(A)** and 14 **(B)** days after transfection with different polyplexes. ALP staining measured at 7 **(C)** and 14 **(D)** days after transfection with different polyplexes. **(E)** Alizarin red S staining measured after 21 days of osteogenic induction. **(F)** Quantitative analysis of alizarin red S staining. Representative images of three independent experiments were shown. Data are presented as the means ± SD of three independent experiments, *N* = 3. ^∗∗^*P* < 0.01.

Human bone mesenchymal stem cells, which were transfected with the PEI25kDa/siRNA and PEI-Et/siRNA polyplex packaging Chordin-specific siRNA, were cultured in osteogenic medium to induce osteogenic differentiation. Alkaline phosphatase was stained and its activity was monitored after 7- and 14-days osteogenic induction. When hBMSCs were transfected with Chordin-specific siRNA at 7 (**Figures 6A,C**) and 14 (**Figures 6B,D**) days post-transfection, ALP activity was significantly enhanced via PEI-Et and PEI25kDa compared with the negative control (naked siRNA) and the PEI800/Chordin-siRNA groups and ALP staining was markedly intense. At 7 and 14 days, the ALP activities of hBMSCs in the PEI-Et/Chordin-siRNA groups were 1.48 and 1.69 times higher than those of the PEI25kDa/Chordin-siRNA groups, respectively. Data gathered from the ALP activity assay and ALP staining indicated that the inhibition of Chordin expression improved hBMSC osteogenic differentiation, and the osteogenic differentiation potentials were higher in the PEI-Et/Chordin-siRNA groups than in the PEI25kDa/Chordin-siRNA groups.

The osteogenic differentiation of hBMSCs leads to calcium nodule formation. An important marker of bone formation is calcium content in mineral deposits in the ECM. Alizarin red staining was conducted to further investigate the potential of hBMSC osteogenic differentiation under inhibited Chordin expression after osteogenic differentiation of hBMSCs for 21 days. The hBMSCs were transfected with PEI-Et/Chordin-siRNA, PEI25kDa/Chordin-siRNA, and PEI800/Chordin-siRNA polyplexes. Alizarin red staining showed that the staining improved when Chordin expression was inhibited, and the staining became deeper in the PEI-Et/Chordin-siRNA and PEI25kDa/Chordin-siRNA groups than in the negative control (naked siRNA) groups and PEI800/Chordin-siRNA (**Figure 6E**). Alizarin red staining could also be quantified using a spectrophotometer with OD548 nm. The results indicated that inhibition of Chordin expression significantly improved the calcium deposition in mineralized nodules and 1.25 times more in the PEI-Et/Chordin-siRNA groups than in the PEI 25 kDa/Chordin-siRNA groups (**Figure 6F**). Alizarin red staining and its quantitative analysis provided sufficient evidence that the inhibited Chordin expression could enhance the osteogenic competence of hBMSCs. Moreover, the effect of PEI-Et/Chordin-siRNA on hBMSCs was stronger compared with that of PEI25kDa/Chordin-siRNA.

### Chordin Knockdown Promotes Bone Regeneration of hBMSCs *In Vivo*

**FIGURE 7:**
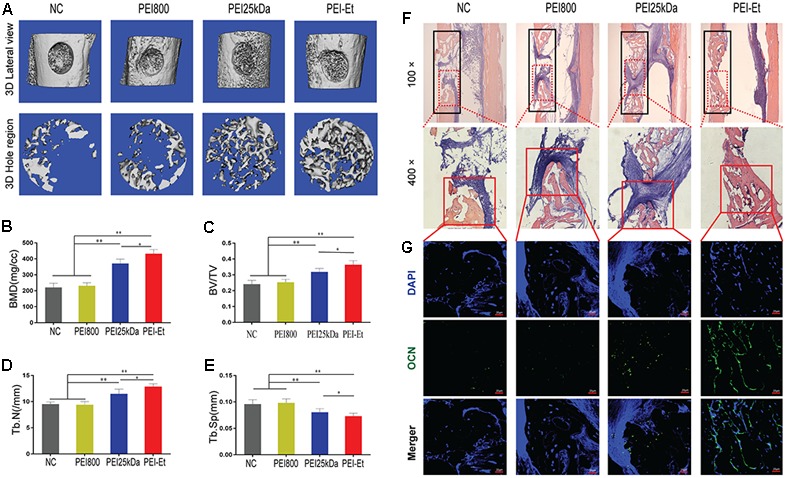
Chordin knockdown promotes the bone regeneration of hBMSCs *in vivo*. **(A)** Lateral views of 3D reconstruction of injured tibiae (Top) and mineralized bone formed in hole region (Lower) by μCT. Representative images from each group, *N* = 5. **(B–E)** 3D structural parameters- BMD, BV/TV, Tb.N and Tb.Sp-of mineralized bone formed in hole region by μCT, *N* = 5. **(F)** Representative images of H&E staining showed new bone formation in injured tibiae (black frame in top panel with 100× and lower panel with 400×). **(G)** Immunofluorescent assay of OCN expression in the new bone. ^∗^*P* < 0.05, ^∗∗^*P* < 0.01.

To investigate the function of Chordin inhibition *in vivo*, we determined the osteogenic activity of hBMSCs transfected with Chordin-specific siRNA by using femoral monocortical defect model. hBMSCs were transfected with Chordin siRNA using PEI-Et, PEI25kDa and PEI800 and were subsequently mixed with Matrigel and transplanted to the osseous hole. μCT was used to quantify the newly formed bone in the defect at 1 month after hBMSCs transplanted. Lateral views of the 3D reconstruction of injured tibia showed higher mineralized tissue in PEI-Et/Chordin-siRNA group than PEI25kDa/Chordin-siRNA group and mineralized tissue in PEI800/Chordin-siRNA and NC group were least (**Figure 7A**). Compared to PEI25kDa/Chordin-siRNA group, bone mineral density (BMD), bone volume density (BV/TV), trabecular number (Tb.N), trabecular thickness (Tb.Th) in PEI-Et/Chordin-siRNA group was increased about 1.16, 1.14 and 1.12 times, respectively (**Figures 7B–D**). In another hand, trabecular separation (Tb.Sp) was reduced by 9.93% in PEI-Et/Chordin-siRNA group compared to PEI25kDa/Chordin-siRNA group (**Figure 7E**). New bone formation is also revealed by H&E staining, and the new bone formation area was higher in PEI-Et/Chordin-siRNA than PEI25kDa/Chordin-siRNA group, however, that was vary small in PEI800/Chordin-siRNA and NC group (**Figure 7F**). The expression of OCN which was specifically expressed in osteoblast using immunofluorescence showed that PEI-Et/Chordin-siRNA group had more osteoblast than PEI25kDa/Chordin-siRNA and other groups in the new bone formation area (**Figure 7G**). These results indicating Chordin knockdown promote bone regeneration of hBMSCs *in vivo*. PEI-Et/Chordin-siRNA polyplexes had more potential in promoting bone regeneration of hBMSCs than PEI25kDa/Chordin-siRNA polyplexes.

## Discussion

With advances in siRNA research, the multi-target and off target issues of siRNA have been resolved through a strict design (Reynolds et al., 2004). However, the lack of safe and effective siRNA vector impedes the application of siRNA technology in clinical treatment (Whitehead et al., 2009). PEI25kDa is a widely reported siRNA delivery vector with highly effective transfection. However, the cytotoxicity of PEI25kDa is too high for clinical application. The main factor affects cytotoxicity, and the transfection efficiency of PEI is the molecular weight of PEI. High-molecular-weight PEI exhibits high transfection efficiency and high cytotoxicity. Low-molecular-weight PEI (<2 kDa) exhibits no cytotoxicity and poor transfection efficiency. To resolve this problem, we designed a cross-linked low molecular PEI by biodegradable biscarbamate linkage named PEI-Et (Mn:1220, Mw:2895). PEI-Et condensed siRNA into about 180 nm sized nanoparticles. PEI-Et showed lower cytotoxicity to hBMSCs compared with PEI25kDa. The former also showed the best transfection efficiency, similar to that of PEI25kDa when the PEI-Et/siRNA N/P ratio was 75. We thus hypothesized that the condensing ability and high transfection efficiency were not attributed to the molecular weight but rather to the specific cross-linked structure of PEI. As for the cytotoxicity, PEI25kDa induced a higher percentage of apoptotic cells than did PEI-Et. The mitochondrial membrane was more severely damaged by PEI25kDa than PEI-Et. The relatively lower molecular weight and degradability of PEI-Et may account for the less severe damage to the mitochondrial membrane. Meanwhile, the PEI-Et group exhibited a higher knockdown efficiency than did the PEI25kDa group. This difference may be attributed to the higher uptake of PEI-Et/Chordin-siRNA polyplex than that of PEI25kDa, as shown in **Figure 4**. In addition, the degradability of PEI-Et may enhanced the lysosome escape of siRNA.

Kwong et al. (2008) and Schneider et al. (2014) reported that Chordin knockdown enhanced the osteogenic of bone marrow and adipose tissue-derived stem cells *in vitro*. Noggin was also a BMP-2 antagonist which was widely studied (Albers et al., 2012; Bernatik et al., 2017). The effect of Noggin on the osteogenic differentiation of BMSCs was bilateral. Inhibited the expression of Noggin in mouse cells could enhanced the osteogenesis *in vitro* and *in vivo* (Wan et al., 2007; Takayama et al., 2009). However, Noggin suppression decreases BMP-2 induced osteogenesis of human bone marrow-derived mesenchymal stem cells *in vitro* (Chen et al., 2012). In the present study, we examined the expression of osteogenic-related gene after Chordin knockdown. The results showed that Chordin knockdown enhanced the BMP-2-Smad1/5/8 signaling pathway, which may be attributed to increased BMP-2 bioavailability. Therefore, suppressed the expression of Chordin could indirectly promote the osteogenic differentiation of hBMSCs through BMP-2-Smad1/5/8 signaling pathway. The effect of Chordin expression on the osteogenic differentiation of hBMSCs depends on the expression of Chordin in hBMSCs and the binding ability of Chordin to BMP-2. If the expression level of Chordin is high and Chordin binds to BMP-2 with high capability, inhibit the expression of Chordin will increase the osteogenic differentiation of hBMSCs significantly. If the expression of Chordin is relatively low, then the impact of Chordin suppression on the osteogenic differentiation may be modest. The current study is the first to report that Chordin knockdown enhanced bone regeneration *in vivo*.

## Conclusion

The nuclear acid carrier PEI-Et with a relatively lower molecular weight (Mn:1220, Mw:2895) exhibits the same siRNA condensation capability as PEI 25 kDa and with biodegradable biscarbamate cross-linker. Owing to these superior characteristics, PEI-Et exhibits relatively lower cytotoxicity and higher transfection efficiency than PEI25kDa. Suppression of Chordin expression improved the osteogenic differentiation of hBMSCs *in vitro* and increased the bone regeneration of hBMSCs *in vivo*. These results indicated that PEI-Et can be used to deliver Chordin-siRNA to hBMSCs and allows the design of novel MSC-based therapies to treat bone fracture healing and bone non-union therapy.

## Author Contributions

CW, WY, FX, TJ, XC, and XlZ: conception and design. CW, WY, FX, YG, XtZ, ZZ, XyZ, PC, and CZ: experiments and/or data analysis. XC: clinical consultancy. XlZ: intellectual input and supervision. CW and FX: article writing with contributions from other authors.

## Conflict of Interest Statement

The authors declare that the research was conducted in the absence of any commercial or financial relationships that could be construed as a potential conflict of interest.

## Supplementary Material

FIGURE S1 Identification and characterization of hBMSCs. **(A)** Representative flow cytometry images of expressions of hematopoietic stem cell marker (CD34), leukocyte marker (CD45) and MSC markers (CD90, CD29, CD44, and CD105) in hBMSCs. **(B)** Adipogenic differentiation potential of hBMSCs *in vitro*. Oil red-O staining was detected in cells after 14 days of culture in adipogenic medium or basal medium (BM). **(C)** Osteogenic differentiation potential of hBMSCs *in vitro*. Alizarin red staining of hBMSCs detected after 21 days of culture in osteogenic medium or BM. **(D)** Chondrogenic differentiation potential of micromass of hBMSCs *in vitro*. The cells were stained with Alcian blue after 14 days of culture in chondrogenic medium or BM.

FIGURE S2 The schematic of interest regions for μCT analysis in femoral monocortical defect model. **(A)** The monocortical osseous hole (0.8 mm diameter) was created on the anterior surface of the femoral crest (red dashed box). **(B)** Cross-section of intermediate position of the bone defect. (i) The hole region between the interrupted cortical bone ends (blue dashed box), (ii) injured BM (red dashed box), and (iii) periosteal callus outside the hole (Yellow dashed box).
