# Supplementary figures and images for: Biscarbamate Cross-Linked Low-Molecular-Weight Polyethylenimine for Delivering Anti-chordin siRNA into Human Mesenchymal Stem Cells for Improving Bone Regeneration
Source: Front Pharmacol. 2017 Aug 28;8:572. doi: 10.3389/fphar.2017.00572 (PMC5609535; doi:10.3389/fphar.2017.00572)

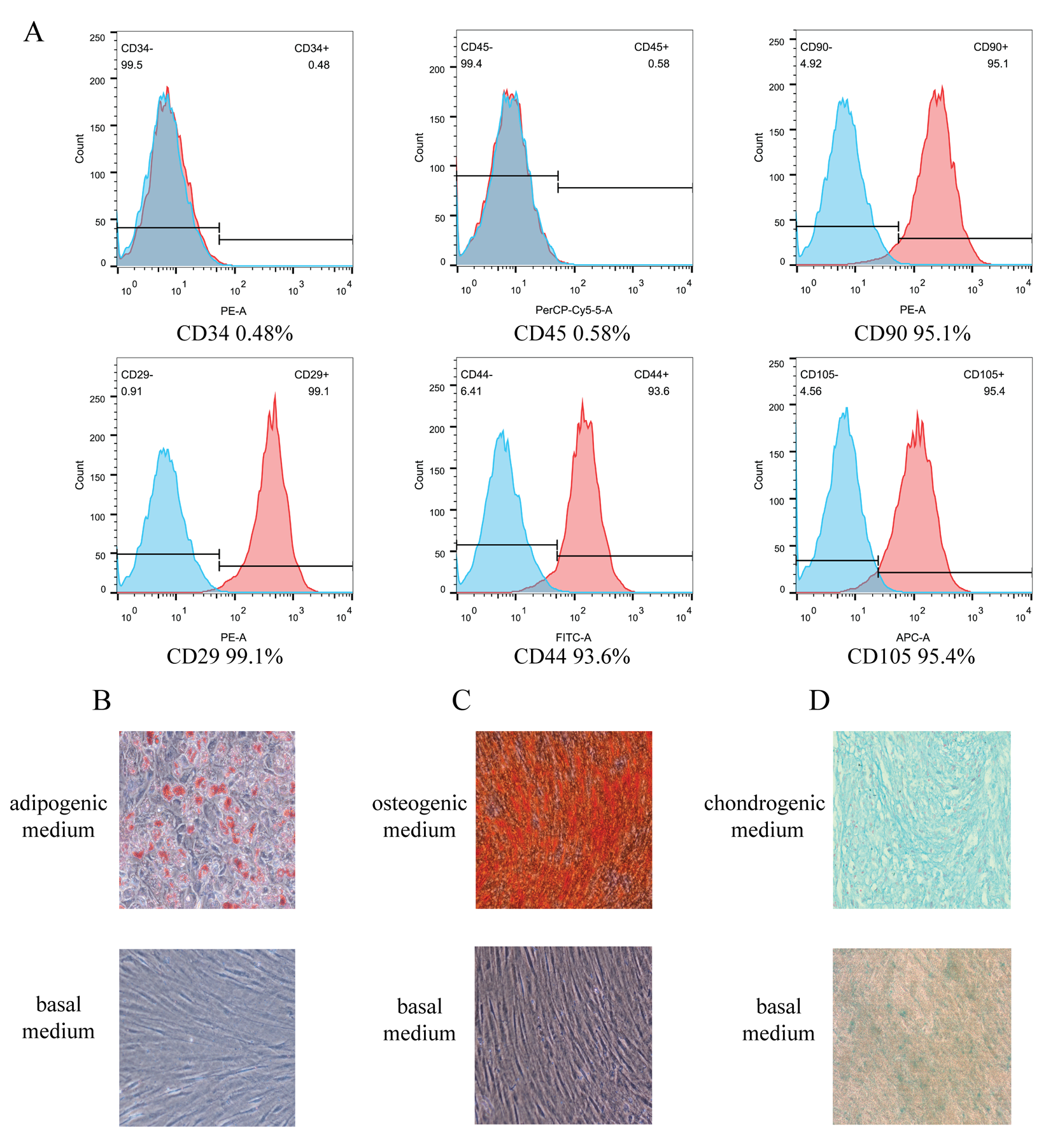

Supplement: FIGURE S1 — Identification and characterization of hBMSCs. (A) Representative flow cytometry images of expressions of hematopoietic stem cell marker (CD34), leukocyte marker (CD45) and MSC markers (CD90, CD29, CD44, and CD105) in hBMSCs. (B) Adipogenic differentiation potential of hBMSCs in vitro. Oil red-O staining was detected in cells after 14 days of culture in adipogenic medium or basal medium (BM). (C) Osteogenic differentiation potential of hBMSCs in vitro. Alizarin red staining of hBMSCs detected after 21 days of culture in osteogenic medium or BM. (D) Chondrogenic differentiation potential of micromass of hBMSCs in vitro. The cells were stained with Alcian blue after 14 days of culture in chondrogenic medium or BM. [file Image_1.TIF]

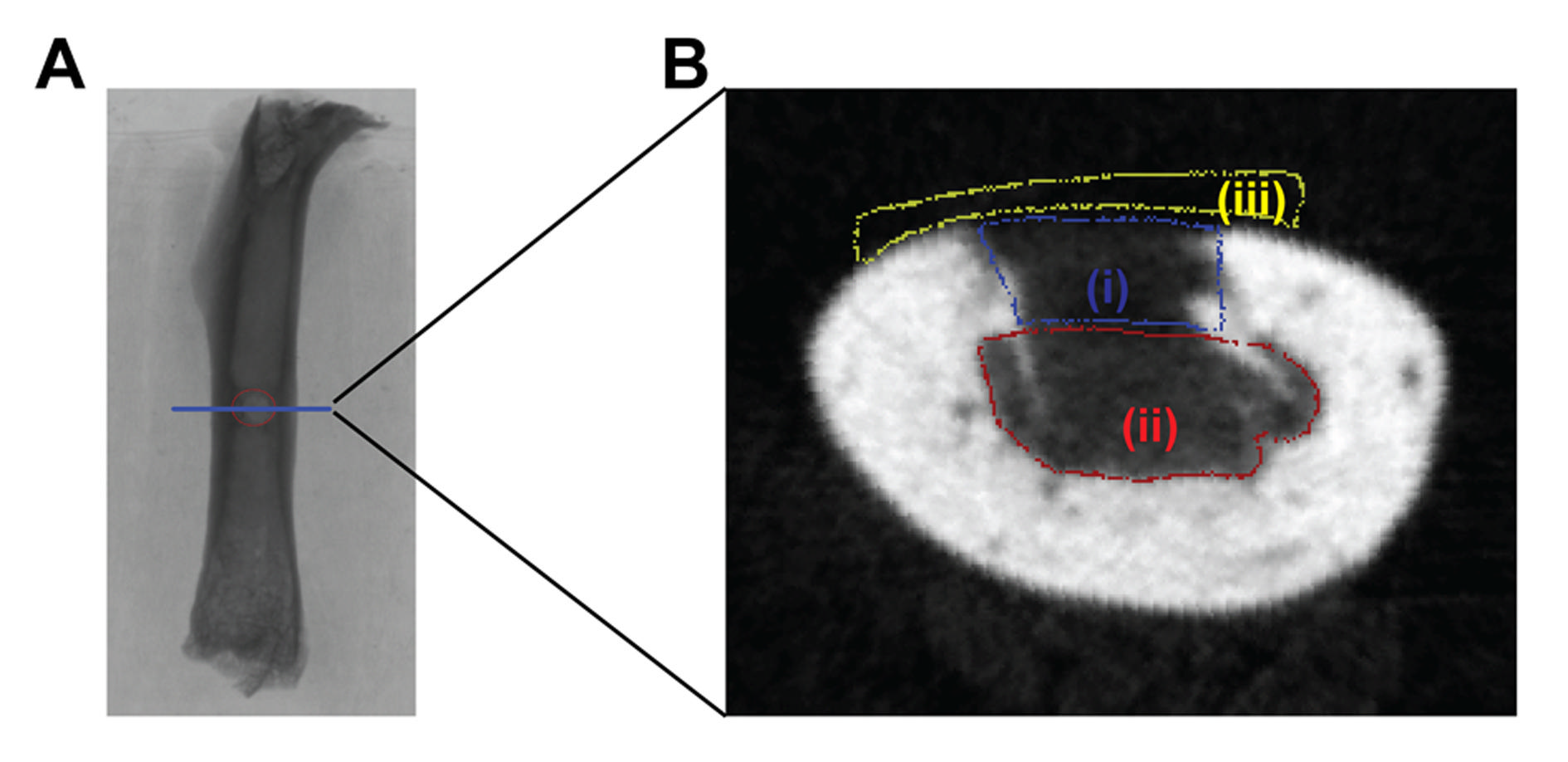

Supplement: FIGURE S2 — The schematic of interest regions for μCT analysis in femoral monocortical defect model. (A) The monocortical osseous hole (0.8 mm diameter) was created on the anterior surface of the femoral crest (red dashed box). (B) Cross-section of intermediate position of the bone defect. (i) The hole region between the interrupted cortical bone ends (blue dashed box), (ii) injured BM (red dashed box), and (iii) periosteal callus outside the hole (Yellow dashed box). [file Image_2.TIF]
